# Profiles of Primary School Children’s Sports Participation and Their Motor Competencies

**DOI:** 10.3390/children11111370

**Published:** 2024-11-12

**Authors:** Johanna Kress, Kathrin Bretz, Christian Herrmann, Patricia Schuler, Ilaria Ferrari

**Affiliations:** 1 Research Group Exercise and Sport, Zurich University of Teacher Education, 8090 Zurich, Switzerland; johanna.kress@phzh.ch (J.K.); kathrin.bretz@phzh.ch (K.B.); christian.herrmann@phzh.ch (C.H.); 2 Centre for Teaching Professions and Continuing Professional Development, Zurich University of Teacher Education, 8090 Zurich, Switzerland; patricia.schuler@phzh.ch

**Keywords:** physical activity, organized sports, informal sports, latent profile analysis, all-day schools, primary school

## Abstract

Background/Objectives: Children participate in various organized and informal physical activities (PAs) in their leisure time, presenting diverse objectives and environments for motor and social development. However, current research often focuses on specific, mostly organized activities, overlooking the complexity of participation across different settings. This study aimed to (1) identify groups of children with similar characteristics based on their participation in five organized and informal sports activities and (2) examine how the groups differ regarding gender, age, BMI, motor competencies (MCs), and attendance in institutionalized care at school. Methods: The study included *n* = 1717 1st and 2nd graders (M = 7.60 years, SD = 0.59, 50.7% girls) and *n* = 1319 3rd and 4th graders (M = 9.46 years, SD = 0.57, 49.4% girls) from the “EMOKK” study, funded by the Swiss National Science Foundation. Data were collected via parent questionnaires on leisure sports participation, and MCs were assessed using MOBAK-1-2 and 3-4 tests. Latent profile analyses (LPAs) and univariate ANOVAs were used to identify group differences. Results: A three-profile LPA model best fits the data, revealing differences in participation across individual and team sports, optional school sports, free play on the school playground, and informal activities during leisure time. Children involved more in team sports (profiles: *allrounder* and *very active sportsperson*) participate more in informal play and present better MCs than children participating mainly in individual sports (profile: *individual sportsperson*). Girls were predominantly in the individual sports profile, while boys were more evenly spread across all groups. These findings highlight the importance of designing targeted interventions that promote participation in both organized and informal sports, particularly for children with lower levels of PA. Conclusions: Children show different patterns of engagement in different interrelated organized and informal leisure PA contexts. These specific patterns and the children’s MCs should be taken into account for the targeted promotion of PAs during leisure time.

## 1. Introduction

In childhood, physical activity (PA), movement, and play are essential for exploring the environment. Through movement, children interact with their environment and other children and learn to estimate their competencies [1]. Both motor competencies and interdisciplinary competencies (e.g., social skills) are developed in social contexts (e.g., sports club), in interactions with peers (e.g., playing during free time), and in Physical Education (PE) [2,3,4]. PA is relevant from a health-related perspective across the entire lifespan [5] and the World Health Organization (WHO) recommends at least one hour of moderate-to-vigorous PA per day for children and adolescents. However, national and international studies show that these PA recommendations are often not met [6,7,8].

However, PA, play, and sports are among the most frequent and popular leisure time activities from the perspective of children and young people. In European countries, around two-thirds of children participate in sports club activities, making these activities the most widespread form of youth organization engagement among children and adolescents [9,10,11]. It is acknowledged that children are particularly active in sports with friends, which further emphasizes the importance of the social setting in sports [12].

In general, PA is influenced by a variety of factors. In the early childhood period, the family context is undoubtedly a significant determinant, given that the family is typically the initial and primary setting for children’s PA [13]. Contextual and socio-cultural factors such as resource availability, cultural values, and family and community support influence the design, implementation, and outcomes of youth sports participation [14,15]. Age, gender, ethnicity, self-concept, and Body Mass Index (BMI) were identified as the most common factors influencing overall participation in PA during childhood [16]. The studies in the review of Hu and colleagues [16] indicated that girls were less active than boys and boys were spending more time in informal, recreational PAs. Additionally, younger children were found to be more active than older children, whereby a decrease in PA with increasing age could be observed. While BMI is frequently indicated as an influencing factor, its relationship with sports participation remains unclear [16,17]. Cairney and Veldhuizen [17] observed a weak bidirectional relationship, indicating that lower BMI predicts future sports participation, and conversely, participation in sports leads to lower BMIs.

The assessment of PA can be carried out in different ways. In contexts such as public health, PA is often measured quantitatively by wearables, e.g., with accelerometers [18]. However, this method only provides information on the quantity of movement, neglecting context-specific PA patterns and the children’s intentions to move. Other studies assess PA through a questionnaire investigating not only the amount but also the quality of PA, movement, and play, paying particular attention to the content and the context-specific objectives of the activities. Considering the approach of PA as the participation in the culture of sport and movement [19], the contexts in which children engage are relevant [20]. This approach focuses on the fact that movement and sports provide children with differentiated experiences and learning opportunities for the development not only of sports-related competencies but also of social and cognitive competencies [21,22]. Indeed, the pedagogical effect and the educational potential of learning opportunities in sports depend on their motives and goals [11,14].

It is therefore pertinent to enquire as to the context and manner in which the children move. Neuber and Golenia [11] developed a model that categorizes PA learning environments into specific settings. This model, which is established in German-speaking countries, identifies eight central sports-related learning contexts—family, daycare center, school, all-day school, sports club, child and youth welfare, informal sports, and commercial sports—for children and adolescents. The learning contexts were distinguished on a continuum between formal (e.g., schools), non-formal (e.g., sports clubs), and informal settings (e.g., informal sports and family) and between formal and informal educational processes. For example, engagement in sports clubs is understood as a formal educational process in a non-formal setting, whereas extracurricular PA activities in all-day schools are seen as an informal educational process in a formal setting. The classifications of the PA contexts in the following study are based on this concept linked to different settings. Additionally, the analyzed contexts were distinguished between organized sports, which refer to club sports or organized extracurricular school sports courses, and informal sports. The latter category encompasses “non-organized” movement and play situations, including free play on school grounds, during institutionalized care at school, or during leisure time whether alone (e.g., cycling) or with friends (e.g., playing football) outside of school or club settings. Understanding the balance between organized and informal sports participation is essential for developing holistic public health interventions that address both structured PE and free play in childhood [15,23]. While organized sports have been widely studied, fewer studies have explored how informal play contributes to overall PA levels and motor development in children, creating a gap that this study aims to fill.

**Organized sports activities in sports clubs** are often a central focus in discussions about PA and movement during leisure time. Indeed sports clubs, as mentioned before, are one of the most important non-formal settings to engage in sports for children [9,10]. Several studies show that boys enroll more often in sports clubs than girls, with around 60–70% of boys participating in sports clubs, while the participation rate of girls is around 50–60% depending on the country [10,12,24]. Girls predominantly participate in aesthetic and individual sports (e.g., gymnastics, dancing, water sports, and track and field) whereas the majority of boys engage in team sports such as football and floorball or racket sports and combat sports [10,25]. Differences in sports club participation were not found for body weight; however, the participation declines during the elementary school years and becomes more pronounced during middle school years [26,27].

The school setting comprises several meaningful non-formal and informal contexts for daily PA in children characterized by an informal learning environment [11,28,29]. In Switzerland, schools offer **organized extracurricular school sports** activities (in this article, it is called optional school sports) that promote sports in the school environment in a non-formal setting, so that accessibility for all children is given. These organized sports opportunities complement the PE classes and serve as a bridge to the sports clubs, enhancing the participants’ enjoyment of specific sports and potentially encouraging their transition into club sports [10].

**Informal sports and PAs** with friends, family, or alone make an important contribution to children’s overall PA and are the second most important setting for engaging in sports, after sports clubs [10]. The school playground offers an important informal setting for PA, as children use it for free play and movement during lunch breaks, but also after school and on the weekends, with other children or family members. Indeed the school environment is particularly found to be closely associated with children’s PA levels [29]. It provides a familiar and accessible space for children to also engage in active outdoor play with other children [30]. In addition, it was found that the presence of active children enhances overall PA levels in playgrounds. However, girls’ PA tends to decrease when boys are present [30].

The **all-day school** setting comprises non-formal and informal contexts such as organized extracurricular school sports as well as informal free play on the school playground during recess or in institutionalized care time at school. As institutionalized care at school is becoming increasingly important in Switzerland [31], all-day schools assume the responsibility for the development of suitable and varied PA and sports programs for children and young people [32]. The program comprises after-school extracurricular sports courses, supervised PA during lunchtime (open gym), and free PA during recess and before and after school [32].

**Motor competencies (MCs)** are a prerequisite to actively participate in all the described contexts and thus in the culture of sports and movement [33]. They are necessary to develop sport-specific skills, which are needed for sustained engagement in sports and for fostering an active lifestyle throughout the lifespan [34]. The motor competencies (MCs) describe the ability to perform a range of motor tasks, including coordinating gross and fine movements that are essential for daily sports activities [35,36,37]. Numerous studies have identified the determinants and influencing factors of MCs. MCs have been shown to be determinants of PA with higher levels of MCs positively correlating with higher levels of PA and better health attributes [38]. MCs are developed through childhood and adolescence and are dependent and influenced by different biological (e.g., sex and BMI), and environmental factors (e.g., participation in learning situations or opportunities to play) and their interplay [34,39]. Children participating in sports clubs show better MCs in object control than children not participating [33]. In this article, MCs will be used as an umbrella term encompassing the construct of basic motor competencies as a subset of MCs.

Overall, it can be concluded that leisure sports activities occur within a range of learning environments and settings, with varying goals and characteristics. However, a comprehensive overview of participation in different contexts in PA has yet to be established. This study aims to analyze and present the contexts in which children in Switzerland engage in leisure time PA, elucidating the interrelationships between these contexts. A key aspect of this explorative and innovative approach is the analysis of participation profiles, which allows us to identify and highlight the specific characteristics of different groups.

This study aimed (1) to identify groups of children with similar characteristics based on their participation in five organized and informal sports activities and (2) to examine how the groups differ regarding gender, age, BMI, MCs, and attendance in institutionalized care time at school.

## 2. Materials and Methods

The data for this cross-sectional analysis were derived from a study that investigated the “Development of basic motor competencies in children (EMOKK)” (2021–2025) founded by the Swiss National Science Foundation (SNSF; grant number 200840). The MCs of 1st- to 4th-grade primary school children were assessed through the basic motor competency assessment test MOBAK [40], and information, e.g., about the children’s PA, was collected in a proxy parent questionnaire [41].

Additionally, a subsample from a sub-study of EMOKK called “Sports at schools with all-day facilities (SINTA)” (2022–2024) has been included. Data for additional information were collected from two schools in the German-speaking part of Switzerland with a movement-oriented all-day structure (these schools were partly from the EMOKK study and the SINTA sub-study). As part of the SINTA project, the professionals at these schools participated in an in-school training course on PAs and sports in childcare. They were trained in the importance of movement in everyday life and received didactic input on movement in the sports hall. The 3rd- and 4th-grade primary school children were asked about the extracurricular PA programs at the all-day school using a questionnaire; these questions extend beyond the EMOKK study. [42].

The study was conducted in accordance with the principles of voluntary participation, and the children’s legal guardians were duly informed of the study’s objectives and procedures prior to its commencement. The legal guardians provided informed consent, and the children gave their assent to participate. The study fully conforms to the Declaration of Helsinki. The legal and school-relevant ethical requirements were approved by the Ethics Commission of the University of Zurich (No. 21.2.5) as well as by the school principals of the primary schools concerned.

### 2.1. Sample Description

The study involved primary school children from the 1st, 2nd, 3rd, and 4th grades from the German-, Italian- and French-speaking regions of Switzerland. Data were gathered in the spring of 2024. To facilitate the analysis and account for differences in leisure time sports participation between younger and older children, the tested children were divided into two groups. This division is also given by the two different age-related tests used for the assessment of the MCs [43]. The total sample was divided into two groups: one comprising 1st and 2nd graders (sample 1, *n* = 1717) and the other consisting of 3rd and 4th graders (sample 2, *n* = 1319). Sample 2 comprises a subsample that includes 243 children from movement-oriented all-day schools (SINTA schools), which provided additional data on structured sports opportunities and PAs outside of regular school hours. The characteristics of the samples are described in Table 1.

For the inclusion criteria, children were included in the study if data from at least one sports activity during leisure time were recorded.

### 2.2. Instruments and Procedure

#### 2.2.1. Procedure for Data Collection

Information on children’s sports activities during their leisure time such as playing outside or participating in organized sports and general information about the child was gathered from parents through a paper questionnaire. This survey was distributed to parents as a component of the EMOKK study [41]. The MCs of the children were tested during school hours by a test team of the EMOKK study.

In the subsample of the two movement-oriented all-day schools, students were invited to participate in a survey administered online or in paper format. Before completing the questionnaire, which took about 20 min, students received a brief introduction from either the teacher or a research team member of the SINTA project [42].

#### 2.2.2. Indicator Variables

**Individual and team sports frequency**: These variables represent the frequency of participation in individual or team sports in a sports club, and therefore within the context of organized sports [44]. The parents could answer the question if the child was a member of a sports club (no or yes) and if so, tick or write down the type of sport as well as the frequency per week the child was practicing this sport (0–7 times per week). A total of two different sports could be specified. During data processing, the reported sports were categorized into individual (e.g., track and field or swimming) and team (e.g., football or basketball) sports according to the social patterns involved in practicing the sport and based on previous classifications [33,45]. The values for the frequency of participation in team and individual sports were then summed up to produce overall values (range 0–7 days per week).

**Optional school sports:** Participation in optional school sports, characterized as extracurricular, organized sports offered by the school, was reported by the parents in the proxy questionnaire with the question: “How often does your child participate in voluntary sports activities at school?” (range: 0–7 days per week).

**School playground:** The school playground serves as a formal setting where children can engage in free play and movement (informal sports activities). In the questionnaire, it was asked, how many days per week does the child play in the school playground during free time (outside of compulsory school hours)? (range: 0–7 days per week).

Informal sports outside of the school and outside the sports clubs labeled as **days of sport:** In addition to organized sports, non-organized sports in informal settings, referred to as “informal sports” in this article, play an important role in promoting PA [23]. In the questionnaire, the parents were asked: “On how many days per week is your child active in sports (e.g., cycling, football) for at least half an hour in his/her free time (outside school and sports club)?” (range: 0–7 days per week).

The identified profiles in all samples were further analyzed using the variables gender, age, and BMI of the children to identify differences in the distribution within the profiles. The **gender** (girl or boy dichotomy, as the diverse category was not used) and the **age** in months were determined via parents’ or legal guardians’ proxy reporting. The Body Mass Index (**BMI**) was assessed by measuring the height and weight of the participants using a standardized protocol during the on-site visits of the EMOKK study in the school classes [41].

A further considered variable is the attendance in institutionalized care at schools labeled as **school care** attendance; this indicates how many times a week (0 to 5) the child attends the all-day school offering during lunchtime and in the afternoon after school classes. In Switzerland, many educational establishments provide extended educational facilities, also called all-day schools for children before, between, and after compulsory school lessons. These services are provided on a fee-paying basis, with parents able to decide which days their children will attend and for how long.

The children’s **basic motor competencies**, referred to in this article as the general term MCs, were assessed through the MOBAK test instruments [46]. This test instrument is age-specific and demonstrates curricular validity, aligning with the minimal curricular requirements for PE in Switzerland and many European countries [43,47,48]. The MCs were assessed by a research team using the MOBAK 1-2 and 3-4 test instruments during PE lessons at school [43]. The MOBAK test measures basic motor qualifications in two competence domains, “Self-movement” (SM, representing locomotion) and “Object movement” (OM, indicating object control), with each domain tested using four different test items. Children have two attempts for each item, and each attempt is rated on a dichotomous scale according to specific criteria (0 = failed and 1 = successful). The points are summed up per item with a maximum of 2 points possible for the final item score. The item points can be added for each competence domain (SM and OM), with a maximum of 8 points achievable. The total MC score (MC sum) is calculated by summing the scores of the SM and OM domains, with a range from 0 to 16 points [43]. The psychometric quality of the MOBAK tests has been confirmed in several studies using confirmatory factor analysis [40,46].

In addition to the variables already described, in subsample 2, children attending the all-day school care at least once a week indicated in a questionnaire how often they **participated in the open gym** (PA during the lunchtime supervised by the childcare team) offer (never = 0, sometimes = 1, frequently = 2, always = 3 [42]). This variable was answered only by the children of the two schools that were part of the SINTA project. The open gym can be described as an informal learning context in a formal setting [32]. The open gym is a voluntary offer for children who attend the all-day school structures, which take place at lunchtime or in the afternoon after school. It is supervised by childcare professionals and is voluntary. For this offer, the childcare professionals were trained within the in-house training of the SINTA project. The children have the flexibility to decide each day whether they want to attend the open gym or participate in other leisure time activities such as free play, library, etc.

### 2.3. Data Analysis

This study used a person-centered approach, employing latent profile analysis (LPA) with MPlus version 8 [49]. LPA is a statistical method used to identify different subgroups within a population that share some similar observable characteristics [50]. LPA was selected due to its ability to model heterogeneity within the population and identify latent subgroups based on unobserved patterns of participation in PA. The aim was to identify homogeneous groups of school children with different profiles of sports behaviors based on their frequency of (1) individual and (2) team sports, (3) frequency of participation in optional school sports, (4) movement and play in the school playground, and (5) informal PA during leisure time (variables ranged from 0 to 7 days per week). We applied LPA using five classification variables related to PA in leisure time for the younger primary children in the 1st and 2nd grades, as well as for older children in the 3rd and 4th grades. All the steps of data analysis were carried out analogously for the two age samples. The missing data were handled using full information maximum likelihood (FIML).

The LPA model testing process started with the estimation of a one-profile model, progressively adding more profiles until the optimal number of latent profiles was determined. This process aimed at achieving the best solution both statistically and in terms of theoretical interpretability. The determination of the optimal number of profiles was based on the recommendations of Geiser [51] and of Weller and colleagues [50] following the criteria of the following:

- Model fit: The Bayesian information criterion (BIC), the Akaike information criterion (AIC), and the sample-size-adjusted Bayesian information criterion (SABIC) were reported as indicators of model fit. Lower values indicate a better fit of the model [51,52]. In the fit statistics, the Vuong–Lo–Mendell–Rubin adjusted likelihood ratio test (LMR LR), the Lo–Mendell–Rubin adjusted LRT test (ALMR LR), and the bootstrapped likelihood ratio test (BLRT) provide indications if one model is statistically better than another through a *p*-value [50]. Also, the average latent profile posterior probability that indicates the average probability of an individual being assigned to a specific group is desirable to be high and closer to 1.0. Among some researchers, the cutoff value of 0.80 is accepted, whereas a value greater than 0.90 is considered as ideal. Probabilities between 0.80 and 0.90 are acceptable if other criteria are satisfied and the model is theoretically justified [50]. The entropy was considered for the accuracy determination of group classification. This value was considered acceptable above 0.80 and ideal close to 1 [50].
- Profile size: The sample size per profile was evaluated and models with profiles of <5% were inspected, as they may be spurious [53].
- Interpretability: According to the research questions, the number of profiles should be theoretically meaningful and interpretable [50,51].
- Parsimony: Models with fewer profiles should be preferred to avoid local likelihood maxima and overfitting and to ensure that the profiles can be explained in a satisfactory and meaningful way [51].

It is necessary to point out that the optimal number of profiles was chosen considering both the statistical model fits in conjunction with the theoretical interpretation.

After determining the optimal LPA profile, we compared them to determine differences in gender, age, BMI, attendance in school care, and basic motor competencies in the two competence domains “Self-movement” and “Object movement”. For subsample 2, the covariate of the participation in the open gym was additionally analyzed. We calculated one-way ANOVAs with the covariate variables for group comparisons and descriptive statistics using SPSS 28 [54].

## 3. Results

### 3.1. Latent Profiles of Sports Participation

The LPA identified three distinct profiles of sports participation across both age groups: *individual sportsperson*, *allrounder*, and *very active team sportsperson*. These profiles reveal significant gender differences and associations with motor competencies.

The model fit outcomes of the LPA are shown in Table 2 for grades 1 and 2 and in Table 3 for grades 3 and 4. The aim of the LPA was to identify groups of children sharing similar observable characteristics. In both cases, the AIC, BIC, and ABIC decrease consistently as the number of profiles increases. The log-likelihood was always replicated except for profile 5 for the 3rd and 4th grades. The three-profile solution was chosen as the best fit for the data as the *p*-values of LMR LR, ALMR LR, and BLRT are significant and therefore represent a statistically better model. The average probability of class membership in both samples was over 0.90 (ranging from 0.885 to 0.985), which is more than acceptable. In both profiles, the entropy is >0.80 indicating good accuracy. The profiles contain more than 5% except for one profile in sample 1 which has 4%; nevertheless, it is acceptable, as the other statistical values are well fitting, and the model can be theoretically explained. Compared to the three-profile solution, the four- and five-group solution of sample 1 presents no significant *p*-values in LMR, LR, and ALMR LR, and one profile contains 2% of people. In sample 2, the main reason for not choosing a solution of four or five profiles was the small sample proportion of some profiles (1% and 2% of people) and the theoretical interpretation. Furthermore, the optimal log-likelihood could not be replicated with the five-profile solution.

The analysis identified three different profiles in both samples 1 and 2. The results of the LPAs are described based on sample 2 for grades 3 and 4. The resulting three profiles of sample 2 closely resemble those identified in sample 1. Specific characteristics of sample 1 will be discussed in a subsequent section.

**Figure 1:**
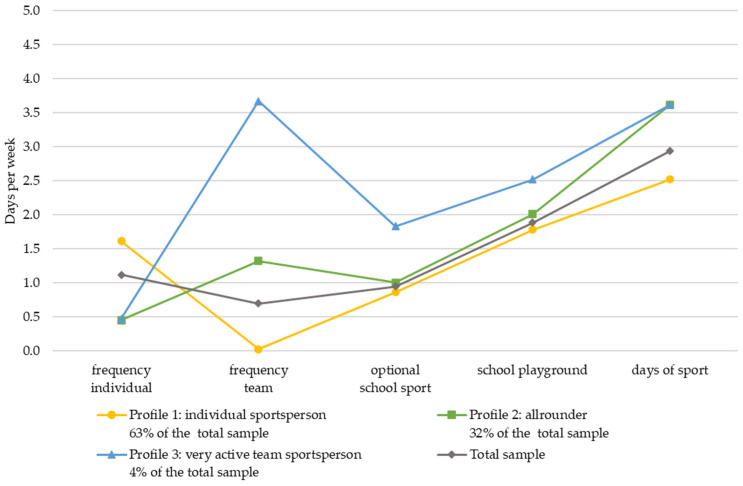
Latent profile results: mean scores of physical activity settings (range: 0–7 days a week) for children of the 1st and 2nd primary school class.

**Figure 2:**
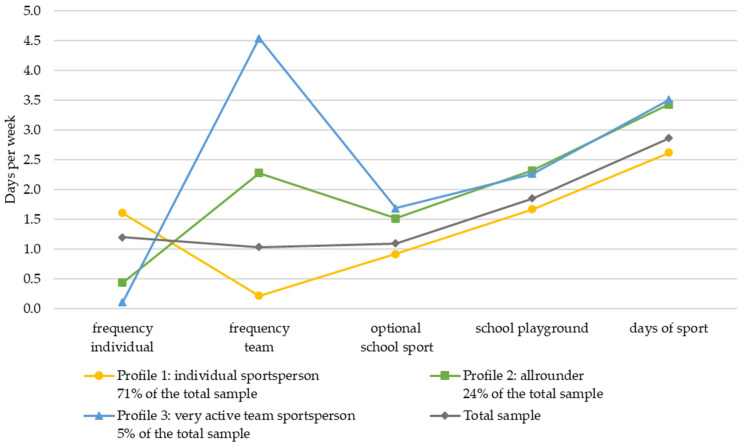
Latent profile results: mean scores of physical activity settings (range: 0–7 days a week) for children of the 3rd and 4th primary school class.

The mean values and standard deviations of the study variables for each group are shown in Table 4 for sample 1 and Table 5 for sample 2. To facilitate the interpretation, the mean values of the three-profile model (lines in colours yellow, green and blue) and for the total sample (gray line) are graphically represented in Figure 1 and Figure 2 showing the five variables included in the LPA on the *x*-axis. On the *y*-axis, the number of days per week (0–7 for all variables) is marked. 

Profile 1 (yellow), labeled as *individual sportsperson*, includes 71% of the majority of sample 2 and is characterized by moderate participation in individual sports about 1.61 times per week and almost no engagement in team sports (M = 0.22 days a week). Participation in optional school sports is moderate but lower than in the other two profiles (M = 0.92). Children in this group show medium-level data in school playgrounds outside school hours (M = 1.67 days a week) and informal PA outside school and club sports (days of sport = 2.62) compared to the other profiles. Comparing the values of the children in this profile with the overall means of the sample, it turns out that these children participate more in individual sports and far less in team sports and engage slightly less than the average in informal sports (namely school sports and days played) and the optional sports at school.

Profile 2, (green) labeled *allrounder*, represents 24% of sample 2. Children in this profile present low participation in individual sports (M = 0.44 days per week), moderate participation in team sports in a club (M = 2.28 days per week), and frequent participation in optional school sports (M = 1.52 days per week). Additionally, they have high levels of informal sports at school (school playground = 2.32 days a week) and even higher levels in settings outside the school (days of sport = 3.43). Comparing the mean values of this profile with the overall mean values (gray line), in the sports contexts, it is noticeable that these children are more active than the average in informal PA contexts during the week and participate more often in team sports in clubs. On the other hand, they practice considerably fewer individual sports than the total average.

In profile 3, (blue) labeled as a *very active team sportsperson*, 5% of sample 2 is included. Characteristic of this profile is that they have minimal participation in individual sports (M = 0.11 days per week) but a remarkably high level of participation in team sports in the club 4.54 times a week. Children in this profile also often participate in optional school sports (M = 1.69 days per week) and informal sports such as free play after school hours at the school playground (M = 2.27) and outside of school and the sports club (M = 3.51). Values in the informal sports activities, namely the school playground and the days of sports of this profile are comparable with the values of profile 1 for these variables and are reasonably higher than the participation of the overall sample. Also, the average participation frequency of children in team sports in this profile is much higher (3.5 days a week) than the overall average values. In contrast, children represented by this profile show almost no participation in individual sports whereas the mean overall value is about 1.2 times a week. Overall, it can be seen that the variables that determine the profiles differ significantly between the profiles (see F-value of the one-way ANOVA in Table 4 and Table 5 in the first half).

When comparing the LPAs of sample 2 of 3rd and 4th grades and sample 1 of 1st- and 2nd-grade children, it can be seen that the calculated three profiles are similar, and the sample proportion per profile is quite comparable (Table 2 and Table 3 and Figure 1 and Figure 2). Nevertheless, the LPAs of the two samples reveal discrepancies, particularly concerning the context of organized sports in a club (as illustrated by a comparison of Figure 1 and Figure 2). Children in the 3rd and 4th grades specialize more in terms of the type of sport, which is particularly evident in the greater differentiation between team and individual sports participation between the different profiles. Children at this age participate at a higher frequency, especially in team sports, than younger children in grades 1 and 2. However, it is remarkable that in both samples, children participating mainly in team-oriented organized sports in the club are also more active in informal PA and sports (school playground and days of sport) than children mainly participating in individual organized club sports.

### 3.2. Characteristics and Differences by Gender, Age, and MCs Between the Profiles

The second half of Table 4 and Table 5 present the descriptive statistics for the variables gender, age, BMI, school care, and the MCs (indicated with MOBAKs) for each profile in the two samples. To compare the three profiles with each other, F-values for age, BMI, school care, and the MCs are indicated, and for gender, chi-squared values are provided. In both samples, significant differences between the three profiles were found for gender (1 = male; 2 = female), age, and the MCs, especially regarding the competence domain OM. The majority of girls are in profile 1 (sample 1 = 83%; sample 2 = 91%), while younger boys are mostly represented by profiles 1 and 2 and older boys are more represented in profile 1. Regarding age, children in profile 1 are significantly younger than in profile 2 in both samples. Concerning MCs, the competencies of children in profiles 2 and 3 (allrounders and very active team sportspersons) are significantly higher than those of children in profile 1 (individual sportspersons) in both analyzed samples. These differences are mainly due to different competence levels in the competence domain of OM.

With respect to children’s BMI, the three profiles in both samples did not significantly differ from each other. However, differences emerged concerning children’s participation in all-day school care. In sample 2, children categorized under profile 2 (allrounder) show a higher frequency of school care attendance compared to those in the other two profiles. This pattern was not found among children in grades 1 and 2 (sample 1). For the analyzed subsample 2 of children attending the two movement-oriented all-day care structures, the children in different profiles did not differ regarding the frequency of participation in the open gym.

## 4. Discussion

The aim of this explorative study was to identify groups of children with similar characteristics based on their participation in five organized and informal sports activities (first research question). The results indicate that children significantly differ in their leisure time sports participation in different contexts. For the two samples of primary school children in years 1 and 2, and years 3 and 4, three different profiles of participation were distinguished. For both age groups, the children showed similar patterns within the profiles, although there were some differences in the levels of participation. The main differentiating factor for the profiles is participation in organized club sports, categorized into team and individual sports. Some children participate mainly in team sports and less in individual sports, or vice versa. These discrepancies became more pronounced as the children became older.

The first and most common profile was called *individual sportsperson*; children in this group mainly attend individual sports in the context of organized sports. The second profile indicates *allrounders*, characterized by participation in team sports and low-level participation in individual sports. It represents almost one-third of 1st and 2nd graders and a quarter of 3rd and 4th graders. The third profile represents only 4–5% of the total samples and the children represented by this profile are labeled as *very active team sportspersons*. These children show considerably more frequent participation in organized team sports but almost no participation in individual sports.

On the other hand, the levels of participation in extracurricular sports activities and informal sports during leisure time differ significantly between profile 1 and profiles 2 and 3. In particular, children in profile 1 (individual sportsperson) showed significantly less participation in informal sports on the school playground and outside of the school context compared to the other two profiles and to the overall means. Profiles 2 (allrounders) and 3 (very active team sportsperson) were quite active and had values above the mean values of the total sample.

Given that children who engage in individual sports are less active in informal sports (profile 1) and children participating in team sports are more active in informal sports (profiles 2 and 3), it could be assumed that participation in team sports has a leverage effect on informal sports. It stands to reason that informal sports also have a selective component, especially considering the social context in which they are embedded. Children are likely to participate in these PAs only if they demonstrate a certain level of skill or competence, as participation is often contingent upon their perceived ability to “perform” in the social setting [55]. This selective nature is reflected in our analyses, which show differences in participation when comparing the profiles by their MC levels. As shown in previous studies, an association has been found between levels of MCs and sports participation [36,45,56]. While children who mainly participated in team sports were better in “Object movement”, children who mainly participated in individual sports had higher levels of MCs in “Self-movement”. As optional sports courses and free play on the playground are often carried out with balls, it could be that children who play ball sports in the sports club participate more often in informal play and sports.

It is known that participation in team sports positively influences MC in both “Object movement” and “Self-movement”; it could be that the children participate more in informal sports due to their better MCs [57]. It seems that practicing team sports plays an important role in informal sports settings. Encouraging team-based PA may have particular potential to improve children’s MCs and promote higher overall activity levels.

Moreover, children participating in organized sports show higher social relationship skills than children who do not [45]. These social relationship skills are needed to play and participate in sports with other children. Bretz, Strotmeyer, and colleagues [36] showed that especially children in team sports show higher levels of perceived motor competencies. As perceived motor competencies are linked to sports participation [58], the link between team sports participation and participation in informal sports could also be explained via the self-concept.

In addition, family factors have been identified as influencing PAs and MCs. Parental support for their child’s physical activity is positively associated with the child’s MCs and PAs [59,60]. Furthermore, children whose parents are physically active show higher PA levels than children with less active parents [61].

In consideration of the second research question—to examine how the profiles differ regarding gender, age, BMI, MCs, and attendance in institutionalized care time at school—it was notable that there were considerable discrepancies between the profiles concerning gender, the MCs, and age. Conversely, in the case of BMI and participation in institutionalized care, there were fewer or no noteworthy differences between the profiles. The weight status (BMI) was found to not be related to the PA behavior and the identified profiles of the children. This finding is in line with the current study situation, in which the significance of BMI for sports participation remains unclear [16]. Gender-specific differences in the distribution of the profiles could be due to socialization effects, which also show themselves in different levels of MCs [56]. These gender differences may stem from cultural norms and societal expectations that associate team sports with boys and individual sports with girls. Targeted programs that challenge these norms could promote more balanced participation across all genders. Age effects are shown to be more important within younger children (1st and 2nd graders) and less in older children (3rd and 4th graders). Older children within both samples seem to participate more in team sports (profiles 2 and 3) than younger children who tend to participate in individual sports and are less active (profile 1).

In the school setting, there are several contexts for PAs that are used by the children. The organized extracurricular optional sports offered by the schools are attended more by children playing team sports (profiles 2 and 3) than by those playing individual sports (profile 1). It should be examined in more depth which topics and sports these courses mainly cover, in order to analyze whether the differences are related to the content of the courses and the children’s interests, or whether the children who are shown with the individual profile (profile 1) are generally less active and do not participate. However, this assumption would be in contrast to previous findings, which show that optional school sports courses also reach less active children than the club spots and slightly more girls than in other organized sports [10]. Also, the school playground seems to make an important contribution to the informal sports participation of the children. As other studies point out, public and school playgrounds are important contexts required to enable informal PA and play for children in a safe and accessible social and physical environment [30]. However, even in this context, children represented by the individual sportspersons (profile 1) are less active than those represented by the two team sports profiles. One reason could be that girls are mainly represented by this profile, and based on the findings of Reimers and colleagues [30], girls’ PAs seem to be suppressed when boys are present in the playground area. The reasons for these discrepancies should be further investigated to better promote and enable sports activities for all children in this context.

Based on the results of this study, the all-day schools can provide a formal setting for informal PA which does not stand in competition with the participation in traditional sports clubs after school, as it was argued about in these contexts [62,63], but it seems to be complementary. In particular, it turned out that older children (3rd and 4th graders) represented by the profile *allrounder* (profile 2) are also more likely to attend all-day school, although the small group of children participating extremely often in team sports (profile 3) attend less the institutionalized care time at school, which would be consistent with the inconclusive results of the actual studies [64]. However, in the subsample of the two movement-oriented all-day schools, children attending the open gym did not show significant differences between the allocation to the profiles. This indicates that all children, independent of their PA behaviors participate in this context. Therefore, the school setting, especially during institutionalized care time at school, could provide a specific context for participation in PA, for example, by promoting low-threshold PA in movement-oriented activities in the informal context, such as by setting up the offer of an open gym. This finding could be used to promote additional non-formal activities in the school setting. According to Telford and colleagues [65], this approach is seen as a means of sustaining PA as children age, making it more enjoyable and accessible. Some studies found that as children grow older, particularly girls, PA levels decline. In line with our findings, researchers suggest promoting informal, non-competitive, and lifestyle-oriented activities that appeal specifically to older girls [65].

Further research on participation in different sports contexts is needed. In this study, it is noteworthy that a considerable proportion of children (about 60% of 1st and 2nd graders and 71% of 3rd and 4th graders) is represented by the *individual sportsperson* (profile 1). The analyses did not identify any profile characterized by generally low participation in all PA contexts. This indicates that most analyzed children participate at least to a certain extent in either organized sports or informal contexts. As the first profile shows lower values in optional school sports and informal sports than the overall mean, it could be assumed that children with lower PA patterns are represented by this profile. Further studies should analyze to which extent different profiles and contexts are related to the overall PA level of children.

Another topic that should be considered in future research about informal sports is the content and the goals of the activities that take place in informal sports. For a better understanding, it would be meaningful to analyze the reasons why children participating mostly in team sports are also more active in informal sports. From a developmental perspective, it would be valuable to examine the ongoing development of sports participation and MCs, as well as their interplay, within a longitudinal research design. Future research should explore longitudinal changes in these activity profiles to assess the long-term impact of early sports participation on physical, social–emotional, and cognitive development.

### Strengths and Limitations

The current state of studies indicates that when it comes to health promotion through leisure time PA, most research focuses on formal, organized programs, while far less attention is given to the effectiveness of informal programs [15]. Nevertheless, some studies indicate the relevance of informal sports in promoting both participation and broader health and social objectives [23,66,67]. This study is distinguished by an innovative, exploratory child-centered approach with LPAs that is dedicated to both the context of organized sports and the non-organized, informal sports in the context of sports in leisure time. The study is based on a substantial database, which enabled the elaboration of movement profiles of children that could be distinguished and described in terms of several characteristics.

In acknowledging the limitations of this study, it is important to consider the situation-specific characteristics of the children’s living environments. As highlighted by Hu and colleagues [16], participation in PA is influenced by cultural contexts and the availability of local opportunities. However, this study’s findings may be constrained by these contextual variables, as the socio-cultural and environmental factors shaping sports participation are locally specific and present different availability and contents (e.g., in the optional school sports or the organized sports in the clubs). Also, the interests and motives of children participating in different contexts are not analyzed in depth. Additionally, the structure of the Swiss school system, where children attend school both in the morning and afternoon, plays a key role in shaping opportunities for PA participation. These aspects should be considered when generalizing the results as these may limit the broader applicability of the findings. Nevertheless, while the study draws from a large sample that includes children from different linguistic regions of Switzerland, this diversity also introduces variability that supports the generalizability of the results. Moreover, it is also important to consider that the children’s participation in different sports contexts was reported via a proxy questionnaire completed by their parents for reasons of practicality.

## 5. Conclusions

This study showed that in the context of leisure time sports and PA, both informal and organized sports are important to consider as both of them contribute significantly to the PA of children. Variations between the identified participation profiles are influenced by multiple factors including gender, age, the type of sport, and especially the MCs. Understanding these differences is crucial for designing comprehensive and effective PA interventions. Accordingly, MCs should also be given special consideration when promoting sports activities in an informal context, as they may have limited or leveraging effects. The findings suggest that different strategies may be needed to promote PA among the found groups. It is recommended to offer different contexts focusing on different contents, in which children have the possibility to move according to their needs and abilities. The school setting may be an important environment that could reach many children, such as the ones who are less active and present lower MCs. The school setting could offer differentiated activities that also consider non-competitive, low-threshold opportunities for PAs and social encounters to provide children with a wide range of extracurricular learning opportunities in the long term and across all age groups.

## Figures and Tables

**Table 1:** Sample description divided by sample 1 (1st and 2nd grades), sample 2 (3rd and 4th grades), and subsample 2 (movement-oriented all-day schools with 3rd and 4th graders).

	Sample 1 1st and 2nd Grade	Sample 2 3rd and 4th Grade	Subsample 2 ^1^ 3rd and 4th Grade
***n***	1717	1319	243
**Age**			
Range (years)	6.4–8.8	8.4–10.8	8.4–10.8
M (years)	7.6	9.5	9.7
SD	0.6	0.6	0.6
**Gender**			
Girls	871 (50.7%)	652 (49.4%)	111 (45.7%)
Boys	846 (49.3%)	667 (50.6%)	132 (54.3%)
Diverse	0 (0%)	0 (0%)	0 (0%)

^1^ 3rd- and 4th-grade children from the two schools with all-day care structures part of the SINTA project. Children from this subsample are also included in the analysis of sample 2.

**Table 2:** Model fit indices for latent profile analysis for 1st and 2nd grades, sample 1.

	AIC	BIC	ABIC	Entropy	LMR LR	ALMR LR	BLRT
1 Profile	26,532.65	26,587.14	26,555.37	-	-	-	-
2 Profiles	25,979.90	26,067.07	26,016.24	0.80	*p* < 0.001	*p* < 0.001	*p* < 0.001
3 Profiles	25,389.64	25,509.50	25,439.61	0.81	*p* < 0.001	*p* < 0.001	*p* < 0.001
4 Profiles	24,327.60	24,480.15	24,391.20	0.85	*p* = 0.101	*p* = 0.105	*p* < 0.001
5 Profiles	23,238.55	23,423.8	23,315.78	0.86	*p* = 0.113	*p* = 0.117	*p* < 0.001

Note: AIC = Akaike information criterion; BIC = Bayesian information criterion; ABIC = sample-size-adjusted BIC; LMR LR = Vuong–Lo–Mendell–Rubin likelihood ratio test; ALMR LR = Lo–Mendell–Rubin adjusted LRT test; BLRT = bootstrap likelihood ratio test.

**Table 3:** Model fit indices for latent profile analysis for 3rd and 4th grades, sample 2.

	AIC	BIC	ABIC	Entropy	LMR LR	ALMR LR	BLRT
1 Profile	22,129.47	22,181.32	22,149.55	-	-	-	-
2 Profiles	21,637.94	21,720.89	21,670.07	0.71	*p* = 0.069	*p* = 0.072	*p* < 0.001
3 Profiles	21,285.10	21,399.16	21,329.28	0.83	*p* = 0.029	*p* = 0.031	*p* < 0.001
4 Profiles	21,005.59	21,150.75	21,061.81	0.85	*p* < 0.001	*p* < 0.001	*p* < 0.001
5 Profiles	20,626.06	20,802.34	20,694.34	0.86	*p* = 0.785	*p* = 0.788	*p* < 0.001

Note: AIC = Akaike information criterion; BIC = Bayesian information criterion; ABIC = Sample-size adjusted BIC; LMR LR = Vuong–Lo–Mendell–Rubin likelihood ratio test; ALMR LR = Lo–Mendell–Rubin adjusted LRT test; BLRT = bootstrap likelihood ratio test.

**Table 4:** Descriptive values of the study’s variables by profile of sample 1 primary school classes 1 and 2.

	Profile 1	Profile 2	Profile 3			
	M CI 95%	M CI 95%	M CI 95%	F	*p*	η2
frequency individual sports *	1.61 [1.55; 1.68]	0.45 [0.39; 0.50]	0.47 [0.24; 0.71]	(2, 1340) = 325.48	<0.001	0.327
frequency team sports *	0.02 [0.01; 0.02]	1.32 [1.28; 1.36]	3.67 [3.47; 3.88]	(2, 1340) = 4628.432	<0.001	0.874
optional school sport *	0.86 [0.8; 0.92]	1.00 [0.91; 1.08]	1.83 [1.38; 2.28]	(2, 1686) = 27.958	<0.001	0.032
school playground *	1.78 [1.67; 1.89]	2.01 [1.84; 2.17]	2.52 [2.06; 2.98]	(2, 1695) = 7.046	<0.001	0.008
days of sport *	2.52 [2.4; 2.64]	3.61 [3.42; 3.80]	3.61 [3.09; 4.14]	(2, 1558) = 53.848	<0.001	0.065
age (in months)	90.78 [90.36; 91.2]	91.69 [91.1; 92.29]	93.27 [91.71; 94.83]	(2, 1714) = 6.442	0.002	0.007
BMI	15.94 [15.81; 16.07]	16.09 [15.89; 16.29]	16.21 [15.77; 16.65]	(2, 1617) = 1.139	0.321	0.001
school care	0.93 [0.84; 1.02]	0.93 [0.8; 1.06]	0.96 [0.56; 1.36]	(2, 1672) = 0.01	0.990	0.000
MC (MOBAKs)	10.08 [9.88; 10.29]	10.83 [10.56; 11.1]	12.06 [11.39; 12.73]	(2, 1495) = 18.907	<0.001	0.025
OM	4.95 [4.84; 5.07]	5.74 [5.59; 5.89]	6.56 [6.23; 6.88]	(2, 1556) = 51.727	<0.001	0.062
SM	5.12 [4.99; 5.25]	5.09 [4.92; 5.27]	5.49 [5.02; 5.95]	(2, 1548) = 1.103	0.332	0.001
				χ2	*p*	
gender (girls)	82.7%	16.2%	1.1%	2 = 284.48	<0.001	
gender (boys)	43.7%	48.6%	7.7%			

* days per week (0–7).

**Table 5:** Descriptive values of the study’s variables by profile of sample 2 primary school classes 3 and 4.

	Profile 1	Profile 2	Profile 3			
	M CI 95%	M CI 95%	M CI 95%	F	*p*	η2
frequency individual sports *	1.61 [1.52; 1.7]	0.44 [0.35; 0.52]	0.11 [0.02; 0.2]	(2, 1080) = 154.686	<0.001	0.223
frequency team sports *	0.22 [0.19; 0.25]	2.28 [2.22; 2.33]	4.54 [4.27; 4.81]	(2, 1080) = 3494.375	<0.001	0.866
optional school sport *	0.92 [0.84; 0.99]	1.52 [1.35; 1.69]	1.69 [1.2; 2.18]	(2, 1305) = 34.3	<0.001	0.050
school playground *	1.67 [1.55; 1.79]	2.32 [2.08; 2.56]	2.27 [1.7; 2.83]	(2, 1298) = 15.022	<0.001	0.023
days of sport *	2.62 [2.48; 2.75]	3.43 [3.19; 3.67]	3.51 [2.98; 4.03]	(2, 1184) = 20.824	<0.001	0.034
age (in months)	113.16 [112.73; 113.6]	114.3 [113.57; 115.03]	113.68 [111.9; 115.47]	(2, 1316) = 3.358	0.035	0.005
BMI	17.45 [17.23; 17.66]	17.04 [16.71; 17.37]	17.54 [16.85; 18.22]	(2, 1228) = 1.962	0.141	0.003
school care	0.62 [0.54; 0.71]	0.80 [0.63; 0.97]	0.36 [0.12; 0.6]	(2, 1282) = 3.534	0.029	0.005
frequency open gym ^a^	0.33 [0.2; 0.46]	0.48 [0.24; 0.72]	0.40 [−0.71; 1.51]	(2, 240) = 0.667	0.514	0.006
MC (MOBAKs)	8.19 [7.96; 8.42]	9.62 [9.25; 9.98]	10.09 [9.26; 10.93]	(2, 1105) = 25.601	<0.001	0.044
OM	3.85 [3.72; 3.99]	5.07 [4.86; 5.27]	5.58 [5.13; 6.03]	(2, 1172) = 56.627	<0.001	0.088
SM	4.31 [4.17; 4.45]	4.58 [4.36; 4.79]	4.47 [4.02; 4.93]	(2, 1153) = 1.915	0.148	0.003
				χ2	*p*	
gender (girls)	91.0%	8.4%	0.6%	2 = 242.153	<0.001	
gender (boys)	52.5%	38.7%	8.8%			

* days per week (0–7); ^a^ data from subsample 2.

## Data Availability

The data will be published in SWISSUbase.ch after the end of the EMOKK project and will be available open access after an embargo period.

## References

[1] Zimmer R. (2020). Handbuch Bewegungserziehung. Grundlagen für Ausbildung und Pädagogische Praxis.

[2] Kirchhoff E., Keller R. (2021). Age-Specific Life Skills Education in School: A Systematic Review. Front. Educ..

[3] Stodden D., Goodway J.D., Langendorfer S.J., Roberton M.A., Rudisill M.E., Garcia C., Garcia L.E. (2008). A Developmental Perspective on the Role of Motor Skill Competence in Physical Activity: An Emergent Relationship. Quest.

[4] World Health Organization (1994). Division of Mental Health Life Skills Education for Children and Adolescents in Schools. Pt. 1, Introduction to Life Skills for Psychosocial Competence. Pt. 2, Guidelines to Facilitate the Development and Implementation of Life Skills Programmes.

[5] Lampert T., Mensink G.B.M., Romahn N., Woll A. (2007). Körperlich-sportliche Aktivität von Kindern und Jugendlichen in Deutschland. Bundesges.—Gesundheitsforsch.—Gesundheitssch..

[6] Guthold R., Stevens G.A., Riley L.M., Bull F.C. (2020). Global Trends in Insufficient Physical Activity among Adolescents: A Pooled Analysis of 298 Population-Based Surveys with 1·6 Million Participants. Lancet Child Adolesc. Health.

[7] Hänggi J., Bringolf-Isler B., Kayser B., Suggs S., Probst-Hensch N. (2022). SOPHYA Studie—Resultate zum Bewegungsverhalten von Kindern und Jugendlichen in der Schweiz.

[8] WHO (2022). Global Status Report on Physical Activity 2022.

[9] Kokko S., Martin L., Geidne S., Van Hoye A., Lane A., Meganck J., Scheerder J., Seghers J., Villberg J., Kudlacek M. (2019). Does Sports Club Participation Contribute to Physical Activity among Children and Adolescents? A Comparison across Six European Countries. Scand. J. Public Health.

[10] Lamprecht M., Bürgi R., Gebert A., Stamm H. (2021). Sport Schweiz 2020: Kinder- und Jugendbericht.

[11] Neuber N., Golenia M., Güllich A., Krüger M. (2018). Lernorte für Kinder und Jugendliche im Sport. Sport in Kultur und Gesellschaft: Handbuch Sport und Sportwissenschaft.

[12] Gerlach E., Herrmann C., Schmidt W., Neuber N., Rauschenbach T., Brandl-Bredenbeck H.P., Süßenbach J., Breuer C. (2015). Effekte Der Sportteilnahme. Dritter Deutscher Kinder- und Jugendsportbericht: Kinder- und Jugendsport im Umbruch.

[13] Richter M. (2008). Soziale Determinanten Der Gesundheit Im Spannungsfeld Zwischen Ungleichheit Und Jugendlichen Lebenswelten: Der WHO-Jugendgesundheitssurvey. Gesundheit.

[14] Bjørndal C., Rudd J. (2024). Systems and Settings for Youth Sport and Physical Education.

[15] Jones G.J., Carlton T., Hyun M., Kanters M., Bocarro J. (2020). Assessing the Contribution of Informal Sport to Leisure-Time Physical Activity: A New Perspective on Social Innovation. Manag. Sport Leis..

[16] Hu D., Zhou S., Crowley-McHattan Z.J., Liu Z. (2021). Factors That Influence Participation in Physical Activity in School-Aged Children and Adolescents: A Systematic Review from the Social Ecological Model Perspective. Int. J. Environ. Res. Public Health.

[17] Cairney J., Veldhuizen S. (2017). Organized Sport and Physical Activity Participation and Body Mass Index in Children and Youth: A Longitudinal Study. Prev. Med. Rep..

[18] Konstabel K., Chopra S., Ojiambo R., Muñiz-Pardos B., Pitsiladis Y., Bammann K., Lissner L., Pigeot I., Ahrens W. (2019). Accelerometry-Based Physical Activity Assessment for Children and Adolescents. Instruments for Health Surveys in Children and Adolescents.

[19] Gogoll A. (2022). Handlungsfähigkeit Und Kompetenzen Im Konzept Der Pragmatischen Sportdidaktik. Schulsport im Spiegel der Zeit.

[20] Demant Klinker C., Schipperijn J., Toftager M., Kerr J., Troelsen J. (2015). When Cities Move Children: Development of a New Methodology to Assess Context-Specific Physical Activity Behaviour among Children and Adolescents Using Accelerometers and GPS. Health Place.

[21] Martins C., Valentini N., Webster K., Carvalho Nobre G., Robinson L., Duncan M., Ribeiro Bandeira P., Barnett L. (2024). Motor Competence as Key to Support Healthy Development of 3-to 5-Year-Old Children: An Expert Statement on Behalf of the International Motor Development Research Consortium. J. Mot. Learn. Dev..

[22] Weiss M.R. (2011). Teach the Children Well: A Holistic Approach to Developing Psychosocial and Behavioral Competencies Through Physical Education. Quest.

[23] Jeanes R., Spaaij R., Penney D., O’Connor J. (2019). Managing Informal Sport Participation: Tensions and Opportunities. Int. J. Sport Policy Polit..

[24] Vilhjalmsson R., Kristjansdottir G. (2003). Gender Differences in Physical Activity in Older Children and Adolescents: The Central Role of Organized Sport. Soc. Sci. Med..

[25] Peral-Suárez Á., Cuadrado-Soto E., Perea J.M., Navia B., López-Sobaler A.M., Ortega R.M. (2020). Physical Activity Practice and Sports Preferences in a Group of Spanish Schoolchildren Depending on Sex and Parental Care: A Gender Perspective. BMC Pediatr..

[26] Drenowatz C., Greier K., Ruedl G., Kopp M. (2019). Association between Club Sports Participation and Physical Fitness across 6- to 14-Year-Old Austrian Youth. Int. J. Environ. Res. Public Health.

[27] Zahner L., Muehlbauer T., Schmid M., Meyer U., Puder J.J., Kriemler S. (2009). Association of Sports Club Participation with Fitness and Fatness in Children. Med. Sci. Sports Exerc..

[28] Bailey R., Ries F., Scheuer C. (2023). Active Schools in Europe—A Review of Empirical Findings. Sustainability.

[29] Broekhuizen K., Scholten A.-M., de Vries S.I. (2014). The Value of (Pre)School Playgrounds for Children’s Physical Activity Level: A Systematic Review. Int. J. Behav. Nutr. Phys. Act..

[30] Reimers A.K., Schoeppe S., Demetriou Y., Knapp G. (2018). Physical Activity and Outdoor Play of Children in Public Playgrounds—Do Gender and Social Environment Matter?. Int. J. Environ. Res. Public Health.

[31] Chiapparini E., Schuler P., Kappler C. (2016). Pädagogische Zuständigkeiten in Tagesschulen. Diskurs Kindh.—Jugendforsch..

[32] Naul R., Neuber N., Neuber N. (2021). Sport im Ganztag—Zwischenbilanz und Perspektiven. Kinder- und Jugendsportforschung in Deutschland—Bilanz und Perspektive.

[33] Herrmann C., Heim C., Seelig H. (2017). Diagnose Und Entwicklung Motorischer Basiskompetenzen. Z. Entwicklungspsychol. Pädagog. Psychol..

[34] Hulteen R.M., Morgan P.J., Barnett L.M., Stodden D.F., Lubans D.R. (2018). Development of Foundational Movement Skills: A Conceptual Model for Physical Activity Across the Lifespan. Sports Med..

[35] Almeida G., Luz C., Rodrigues L.P., Lopes V., Cordovil R. (2023). Profiles of Motor Competence and Its Perception Accuracy among Children: Association with Physical Fitness and Body Fat. Psychol. Sport Exerc..

[36] Bretz K., Strotmeyer A., Seelig H., Herrmann C. (2024). Development and Validation of a Test Instrument for the Assessment of Perceived Basic Motor Competencies in First and Second Graders: The SEMOK-1-2 Instrument. Front. Psychol..

[37] Robinson L.E., Stodden D.F., Barnett L.M., Lopes V.P., Logan S.W., Rodrigues L.P., D’Hondt E. (2015). Motor Competence and Its Effect on Positive Developmental Trajectories of Health. Sports Med..

[38] Barnett L.M., Webster E.K., Hulteen R.M., De Meester A., Valentini N.C., Lenoir M., Pesce C., Getchell N., Lopes V.P., Robinson L.E. (2022). Through the Looking Glass: A Systematic Review of Longitudinal Evidence, Providing New Insight for Motor Competence and Health. Sports Med..

[39] Lopes L., Santos R., Coelho-e-Silva M., Draper C., Mota J., Jidovtseff B., Clark C., Schmidt M., Morgan P., Duncan M. (2021). A Narrative Review of Motor Competence in Children and Adolescents: What We Know and What We Need to Find Out. Int. J. Environ. Res. Public Health.

[40] Herrmann C., Seelig H. (2017). Structure and Profiles of Basic Motor Competencies in the Third Grade-Validation of the Test Instrument MOBAK-3. Percept. Mot. Ski..

[41] Herrmann C., Bretz K., Kress J., Seelig H. (2024). Development of Basic Motor Competencies During Childhood (EMOKK). Documentation of Items and Scales: Survey 2024.

[42] Ferrari I., Schuler P., Kress J. (2024). Sport in Schulen mit Tagesstrukturen (SINTA). Dokumentation der Erhebungsinstrumente.

[43] Hermann C. (2018). MOBAK 1–4: Test zur Erfassung Motorischer Basiskompetenzen für die Klassen 1–4.

[44] Booth V.M., Rowlands A.V., Dollman J. (2015). Physical Activity Temporal Trends among Children and Adolescents. J. Sci. Med. Sport.

[45] Kress J., Seelig H., Bretz K., Ferrari I., Keller R., Kühnis J., Storni S., Herrmann C. (2023). Associations between Basic Motor Competencies, Club Sport Participation, and Social Relationships among Primary School Children. Curr. Issues Sport Sci. CISS.

[46] Herrmann C., Gerlach E., Seelig H. (2015). Development and Validation of a Test Instrument for the Assessment of Basic Motor Competencies in Primary School. Meas. Phys. Educ. Exerc. Sci..

[47] D-EDK Lehrplan 21: Bewegung Und Sport 2017. https://zh.lehrplan.ch/.

[48] Wälti M., Sallen J., Adamakis M., Ennigkeit F., Gerlach E., Heim C., Jidovtseff B., Kossyva I., Labudová J., Masaryková D. (2022). Basic Motor Competencies of 6- to 8-Year-Old Primary School Children in 10 European Countries: A Cross-Sectional Study on Associations With Age, Sex, Body Mass Index, and Physical Activity. Front. Psychol..

[49] Muthén L.K., Muthén B.O. (2017). Mplus User’s Guide: Statistical Analysis with Latent Variables.

[50] Weller B.E., Bowen N.K., Faubert S.J. (2020). Latent Class Analysis: A Guide to Best Practice. J. Black Psychol..

[51] Geiser C., Geiser C. (2011). Latent-Class-Analyse. Datenanalyse mit Mplus: Eine Anwendungsorientierte Einführung.

[52] Ferguson S.L., Moore E.W.G., Hull D.M. (2020). Finding Latent Groups in Observed Data: A Primer on Latent Profile Analysis in Mplus for Applied Researchers. Int. J. Behav. Dev..

[53] Marsh H.W., Lüdtke O., Trautwein U., Morin A.J.S. (2009). Classical Latent Profile Analysis of Academic Self-Concept Dimensions: Synergy of Person- and Variable-Centered Approaches to Theoretical Models of Self-Concept. Struct. Equ. Model..

[54] IBM Corp (2021). IBM SPSS Statistics for Windows.

[55] Barnett L.M., Van Beurden E., Morgan P.J., Brooks L.O., Beard J.R. (2009). Childhood Motor Skill Proficiency as a Predictor of Adolescent Physical Activity. J. Adolesc. Health.

[56] Gramespacher E., Herrmann C., Ennigkeit F., Heim C., Seelig H. (2020). Geschlechtsspezifische Sportsozialisation als Prädiktor motorischer Basiskompetenzen—Ein Mediationsmodell. Motorik.

[57] Herrmann C., Seiler S., Pühse U., Gerlach E. (2017). Motorische Basiskompetenzen in Der Mittelstufe—Konstrukt, Korrelate Und Einflussfaktoren. Unterrichtswissenschaft.

[58] Barnett L.M., Morgan P.J., van Beurden E., Beard J.R. (2008). Perceived Sports Competence Mediates the Relationship between Childhood Motor Skill Proficiency and Adolescent Physical Activity and Fitness: A Longitudinal Assessment. Int. J. Behav. Nutr. Phys. Act..

[59] Laukkanen A., Niemistö D., Finni T., Cantell M., Korhonen E., Sääkslahti A. (2018). Correlates of Physical Activity Parenting: The Skilled Kids Study. Scand. J. Med. Sci. Sports.

[60] Menescardi C., Estevan I. (2021). Parental and Peer Support Matters: A Broad Umbrella of the Role of Perceived Social Support in the Association between Children’s Perceived Motor Competence and Physical Activity. Int. J. Environ. Res. Public Health.

[61] Garriguet D., Colley R., Bushnik T. (2017). Parent-Child Association in Physical Activity and Sedentary Behaviour. Health Rep..

[62] Heim C., Prohl R., Bob A. (2013). Ganztagsschule Und Sportverein. Körper, Bewegung und Schule. Teil 1.

[63] Spengler S., Kuritz A., Rabel M., Mess F. (2019). Are Primary School Children Attending Full-Day School Still Engaged in Sports Clubs?. PLoS ONE.

[64] Neuber N., Züchner I. (2017). Sport in Der Ganztagsschule—Chancen Und Grenzen Für Das Aufwachsen von Kindern Und Jugendlichen. Diskurs Kindh.-Jugendforsch..

[65] Telford A., Salmon J., Timperio A., Crawford D. (2005). Quantifying and Characterizing Physical Activity among 5- to 6- and 10- to 12-Year-Old Children: The Children’s Leisure Activities Study (CLASS). Pediatr. Exerc. Sci..

[66] Lincoln D.J., Clemens S.L. (2021). Where Children Play Sport: A Comparative Analysis of Participation in Organised Sport in School and Club Settings. Health Promot. J. Austr..

[67] Mowen A.J., Baker B.L. (2009). Park, Recreation, Fitness, and Sport Sector Recommendations for a More Physically Active America: A White Paper for the United States National Physical Activity Plan. J. Phys. Act. Health.

